# Episodic stimulation of central chemoreceptor neurons elicits disordered breathing and autonomic dysfunction in volume overload heart failure

**DOI:** 10.1152/ajplung.00007.2019

**Published:** 2019-10-16

**Authors:** Hugo S. Díaz, David C. Andrade, Camilo Toledo, Katherin V. Pereyra, Karla G. Schwarz, Esteban Díaz-Jara, Claudia Lucero, Alexis Arce-Álvarez, Harold D. Schultz, Josiane N. Silva, Ana C. Takakura, Thiago S. Moreira, Noah J. Marcus, Rodrigo Del Rio

**Affiliations:** ^1^Laboratory Cardiorespiratory Control, Department of Physiology, Pontificia Universidad Católica de Chile, Santiago, Chile; ^2^Centro de Investigación en Fisiología del Ejercicio (CIFE), Universidad Mayor, Santiago, Chile; ^3^Centro de Envejecimiento y Regeneración (CARE-UC), Pontificia Universidad Católica de Chile, Santiago, Chile; ^4^Department of Cellular and Integrative Physiology, University of Nebraska Medical Centre, Omaha, Nebraska; ^5^Department of Pharmacology, Institute of Biomedical Sciences, University of São Paulo, São Paulo, Brazil; ^6^Department of Physiology and Biophysics, Institute of Biomedical Sciences, University of São Paulo, São Paulo, Brazil; ^7^Department of Physiology and Pharmacology, Des Moines University, Des Moines, Iowa; ^8^Centro de Excelencia de Biomedicina de Magallanes (CEBIMA), Universidad de Magallanes, Punta Arenas, Chile

**Keywords:** breathing disorders, chemoreflex, heart failure, retrotrapezoid nucleus, ventilatory plasticity

## Abstract

Enhanced central chemoreflex (CC) gain is observed in volume overload heart failure (HF) and is correlated with autonomic dysfunction and breathing disorders. The aim of this study was to determine the role of the CC in the development of respiratory and autonomic dysfunction in HF. Volume overload was surgically created to induce HF in male Sprague-Dawley rats. Radiotelemetry transmitters were implanted for continuous monitoring of blood pressure and heart rate. After recovering from surgery, conscious unrestrained rats were exposed to episodic hypercapnic stimulation [EHS; 10 cycles/5 min, inspiratory fraction of carbon dioxide (${\text{F}I}_{{\text{CO}}_{\text{2}}}$) 7%] in a whole body plethysmograph for recording of cardiorespiratory function. To determine the contribution of CC to cardiorespiratory variables, selective ablation of chemoreceptor neurons within the retrotrapezoid nucleus (RTN) was performed via injection of saporin toxin conjugated to substance P (SSP-SAP). Vehicle-treated rats (HF+Veh and Sham+Veh) were used as controls for SSP-SAP experiments. Sixty minutes post-EHS, minute ventilation was depressed in sham animals relative to HF animals (ΔV̇e: −5.55 ± 2.10 vs. 1.24 ± 1.35 mL/min 100 g, *P* < 0.05; Sham+Veh vs. HF+Veh). Furthermore, EHS resulted in autonomic imbalance, cardiorespiratory entrainment, and ventilatory disturbances in HF+Veh but not Sham+Veh rats, and these effects were significantly attenuated by SSP-SAP treatment. Also, the apnea-hypopnea index (AHI) was significantly lower in HF+SSP-SAP rats compared with HF+Veh rats (AHI: 5.5 ± 0.8 vs. 14.4 ± 1.3 events/h, HF+SSP-SAP vs. HF+Veh, respectively, *P* < 0.05). Finally, EHS-induced respiratory-cardiovascular coupling in HF rats depends on RTN chemoreceptor neurons because it was reduced by SSP-SAP treatment. Overall, EHS triggers ventilatory plasticity and elicits cardiorespiratory abnormalities in HF that are largely dependent on RTN chemoreceptor neurons.

## INTRODUCTION

Heart failure (HF) affects more than 20% of the population over 75 yr of age, and its prevalence is expected to double by 2030 (34). Therapeutic management of HF is costly, and prognosis still remains poor as the 5-yr survival rate is ~50% (34). Disordered breathing (3, 11) and autonomic dysfunction (19) are pathophysiological hallmarks of HF, which are associated with deterioration of cardiac function and increased mortality risk (16, 19, 42). Aberrant cardiovascular reflex function, particularly chemoreflexes, is thought to play a crucial role in the development of these autonomic and respiratory disturbances (8, 11, 40). In support of this notion, HF patients display enhanced ventilatory responses to hypoxia and/or hypercapnia (11). Recently, we have shown that volume overload HF rats display enhanced central chemoreflex (CC) gain concomitant with autonomic dysfunction (7, 39). Of note, enhanced central chemoreflex gain was correlated with disordered breathing and augmented sympathetic tone in these animals (39). However, no comprehensive studies addressing a link between central chemoreceptors and cardiorespiratory alterations in volume overload HF have been conducted.

The neurons that mediate the central chemoreflex (CC) are primarily located in the retrotrapezoid nucleus (RTN) on the ventral medullary surface (14, 26, 40). These neurons respond to changes in CO_2_/H^+^ (21, 32) and elicit a reflex cardiorespiratory response characterized by increases in ventilation (26) and sympathetic outflow (28). This cardiorespiratory response results from excitatory projections to presympathetic neurons located in the rostral ventrolateral medulla (RVLM) (28, 35), as well as to the respiratory central pattern generator (rCPG) (21, 32). Additionally, activation of central chemoreceptors triggers respiratory synchronous modulation of sympathetic nerve activity, leading to respiratory-sympathetic coupling (13, 14). Ablation of RTN chemoreceptor neurons significantly diminishes the hypercapnic ventilatory response (HCVR) in conscious rats without affecting basal ventilation (26). Considering that volume overload heart failure rats display enhanced central chemoreflex sensitivity, sympathoexcitation, and ventilatory instability, and that the RTN neurons send projections to the RVLM and rCPG, we hypothesized that RTN chemoreceptor neurons play a role in HF pathophysiology.

It has been proposed that episodic stimulation of the central chemoreceptors occurring during apneas leads to exaggerated sympathetic responses (40) and that breathing disorders in patients with HF are a function of chemoreceptor-mediated hyperventilation and subsequent decreases in Pco_2_ below the apneic threshold (22). Previous work shows that periodic stimulation of CC triggers ventilatory plasticity, characterized by changes in poststimulation normoxic minute ventilation (V̇e) (4, 27). However, the relationship between this phenomenon and disordered breathing patterns is controversial (27), and the effects of episodic stimulation of CC on breathing disorders in HF has not been studied yet. Considering that disordered breathing, sympathoexcitation, and increased HCVR are observed in rats with HF (2, 7, 39, 40), it is plausible that ventilatory plasticity resulting from hypercapnic stimulation contributes to the development and/or exacerbation of these pathophysiological hallmarks. Therefore, in this study we aimed to assess whether ventilatory plasticity, disordered breathing, and autonomic dysfunction were elicited after episodic hypercapnic stimulation in rats with volume overload HF and whether these sequelae were mediated by RTN chemoreceptor neurons.

## MATERIALS AND METHODS

### 

#### Animals.

Twenty-four adult male Sprague-Dawley rats (250 ±12 g) were used for these experiments. Animals were housed in a controlled temperature environment (22–25°C) with a 12-h light-dark cycle and ad libitum access to food and water, in accordance with the guidelines set forth by the American Physiological Society and the European Convention for the Protection of Vertebrate Animals used for Experimental and other Scientific Purposes (Council of Europe No. 123, Strasbourg 1985). All experimental protocols were approved by the Ethics Committee for Animal Experiments of the Pontificia Universidad Católica de Chile. Experiments were performed 8 wk after induction of HF (Supplemental Fig. S1; all Supplemental Material is available at https://doi.org/10.6084/m9.figshare.9275216.v1). At the end of the experimental protocol, all animals were humanely euthanized with an overdose of anesthesia (pentobarbital sodium 100 mg/kg ip).

#### Volume overload heart failure model.

Rats underwent surgery to produce an arteriovenous (A-V) fistula using the needle technique as previously described (2, 7, 39). Briefly, under anesthesia (Isoflurane: 5% for induction; 1.5% for maintenance balanced with O_2_), the inferior vena cava and the abdominal aorta were exposed using a midline incision. Both vessels were clamped caudal to the renal artery and the aortic bifurcation, respectively. The aorta was punctured using an 18-gauge needle and advanced until it perforated the adjacent vena cava. Immediately afterward, a drop of histoacryl glue (BBraun, Germany) was used to seal the aorta at the puncture point. The A-V fistula was confirmed by visualization of bright red arterial blood entering the vena cava through the anastomosis. The peritoneal cavity was closed with absorbable suture (Novosyn 4/0, BBraun, Germany), and the skin was closed with absorbable suture (Novosyn 3/0, BBraun, Germany) and metallic clips (Kent Scientific, Torrington, CT). Postoperative management consisted of administration of 5 mg sc enrofloxacin, 1 mg sc ketoprofen, 5 mL ip saline solution, and 2% topical lidocaine hydrochloride jelly. Sham-operated rats underwent the same anesthesia and surgical procedures without the anastomosis.

#### Ablation of RTN chemoreceptor neurons.

At 4 wk post-HF or Sham surgery, rats were anesthetized (100 mg/kg ketamine and 10 mg/kg xylazine) and fixed to a stereotaxic frame (Supplemental Fig. S1). Bilateral injections of saporin toxin conjugated to substance P (SSP-SAP; 0.6 ng/30 nL; Advanced Targeting Systems, San Diego, CA) into the RTN were administered to destroy chemoreceptor neurons, as previously described (10, 31, 37, 38). SSP-SAP dose was selected based on previous studies showing a ∼50–60% ablation of Phox2b^+^TH^−^ neurons (i.e., chemoreceptor units) in the RTN of rats (10, 31, 37, 38). Facial motoneurons, catecholaminergic and serotonergic neurons, and neurons located in the ventral respiratory column caudal to the facial motor nucleus are not affected by these injections (37). Three separate injections of 30 nL of SSP-SAP were placed 2.4 mm caudal to lambda, 1.8 mm lateral to the midline, and 8.5 mm below the dura mater (10, 31, 37, 38) separated by 200 μm, using a Hamilton syringe (0.5 µL, Sigma, Germany) connected to a 32-gauge injection needle, as previously described (10, 31, 37, 38). Vehicle-operated rats were injected with sterile saline solution. Enrofloxacin (5 mg sc) and ketoprophen (1 mg sc) were administered postsurgery for protection against infection and pain relief, respectively. After surgery, animals were allowed to recover for 2 wk, and physiological experiments were performed 4 wk after SSP-SAP or saline injections (Supplemental Fig. S1).

#### Echocardiography.

At 4 wk post-HF surgery, cardiac function was evaluated under isoflurane anesthesia (5% for induction; 1.5% for maintenance balanced with O_2_) using transthoracic echocardiography. M-mode echocardiography was recorded for quantification of cardiac dimensions in the midpapillary muscle region with the parasternal short-axis view using a SonoaceR3 imaging system (Samsung, Korea). Left ventricular end-systolic diameter (LVESd) and left ventricular end-diastolic diameter (LVEDd) were measured using averaged measurements from 3 consecutive cardiac cycles in accordance with guidelines set forth by the American Society of Echocardiography (20). The left ventricular end-systolic volume (LVESv) and left ventricular end-diastolic volume (LVEDv) were calculated using the Teicholz method (2, 39). The following criteria were used for volume overload HF: ejection fraction ≥50 and end-diastolic volume and stroke volume ≥1.5-fold changes relative to Sham (2, 7, 39). Subsequently, rats were assigned to one of the following experimental groups: Sham+vehicle (Sham+Veh), Sham+SSP-SAP, HF+Veh, and HF+SSP-SAP (Table 1).

**Table 1.** Echocardiographic parameters at 4 wk post-Sham or HF surgery

	Sham+Veh (*n* = 6)	Sham+SSP-SAP (*n* = 6)	HF+Veh (*n* = 6)	HF+SSP-SAP (*n* = 6)
LVEDV, μL	273.70 ± 35.58	300.20 ± 17.90	383.3 ± 27.20*	353.00 ± 10.72
SV, μL	208.10 ± 18.78	236.40 ± 14.77	323.40 ± 31.58*	302.00 ± 31.96*
EF, %	77.67 ± 3.53	72.85 ± 1.10	80.10 ± 6.34	81.18 ± 7.07
FS, %	48.23 ± 3.22	43.40 ± 0.96	49.92 ± 7.82	57.68 ± 12.10

Values are mean ± SE;* n* = 6 rats per group. EF, ejection fraction; FS, fractional shortening; HF, heart failure; LVEDV, left ventricular end-diastolic volume; SSP-SAP, substance P-saporin toxin; SV, stroke volume. One-way ANOVA, followed by Holm-Sidak post hoc analysis.

* *P* < 0.05 vs. Sham+Veh.

#### Blood pressure telemetry implantation and assessment.

At 7 wk post-HF or Sham surgery, rats were anesthetized with 2% isoflurane in O_2_, and a skin incision was made to expose the femoral artery. The tip of a pressure catheter attached to a telemetry transmitter [PA-C40, Data Sciences International (DSI), New Brighton, MN] was guided into the femoral artery, and the transmitter body was placed into a subcutaneous pocket. After surgery the rats received a subcutaneous injection of ketoprofen (1 mg) and enrofloxacin (1 mg). Arterial blood pressure was measured in conscious, freely-moving rats in a whole body plethysmography chamber (Emka Technologies, France) using a radiotelemetry system (DSI). Blood pressure was recorded at a sampling rate of 500 Hz and heart rate was derived from dP/dt of the arterial pressure recordings (5, 7, 39).

#### Ventilation analyses and episodic chemoreceptor stimulation.

Basal ventilation was recorded by unrestrained whole body plethysmography while the rats breathed room air. The input and output flow of the plethysmograph were set to 2.0 L/min (39), and baseline recordings were made for 1 h (prestimulation phase) (8, 15, 24, 39). Respiratory stability at rest was determined by construction of Poincare plots and quantified by analysis of short-term variability (SD1) and long-term variability (SD2) of the breath-to-breath interval variability over 300 consecutive breaths (8, 15, 39). Apneic episodes (cessation of breathing for a duration ≥3 breathing cycles), hypopnoeas (reductions ≥50% in V_T_ amplitude compared with 3 previous normal breaths), sigh frequency (increase ≥50% in V_T_ amplitude), and postsigh apneas (cessation of breathing for a duration ≥3 breathing cycles immediately after the sigh) were averaged during resting breathing, as previously described (8, 15, 39). Apnea, hypopnea, and postsigh apnea duration were quantified as well. Tidal volume (V_T_), respiratory frequency (R_f_), and minute ventilation (V̇e: V_T_ × R_f_) were determined by unrestrained whole body plethysmography and analyzed using ECG auto software (Emka Technologies, France) (5, 39). Ten-second segments of stable ventilation (10 ± 2 valid cycles) were used for analysis. Following baseline, animals were subjected to episodic hypercapnic stimulation (EHS) (10 cycles of 7% CO_2_/21% O_2_ balance N_2_, 5 min, spaced by normoxic periods of 5 min). At the termination of EHS, ventilation was recorded under normoxic conditions for 90 min (poststimulation phase) to determine if this paradigm resulted in ventilatory plasticity. Two days before EHS experiments, chemoreflex gain was analyzed by estimating the hypoxic ventilatory response (HVR), calculated by the slope between inspired fraction of oxygen ($FI_{O_{2}}$) 21% and 10%, and the HCVR, calculated by the slope between inspiratory fraction of carbon dioxide (${\text{F}I}_{{\text{CO}}_{\text{2}}}$) 0.03% and 7%, as previously described (15, 25, 39). HVR and HCVR were measured during 10-min exposures to either hypoxic or hypercapnic gas challenges. Ventilatory variability as well as apnea incidence were also quantified during the post-EHS phase. All recordings were made at an ambient temperature of 25 ± 2°C, as previously described (39).

#### Cardiac autonomic function analysis.

Cardiac autonomic function was assessed by analysis of heart rate variability (HRV) (2, 7, 8, 15, 39) before and after episodic hypercapnic stimulation. We calculated dP/dt from arterial pressure waveforms to calculate heart rate and applied a Kalman smoothing method before visually inspecting HRV in the time domain. Then, estimations of power spectral density of HRV were obtained for a 10-min window using an autoregressive method after Hann windowing with 50% overlap. Cut-off frequencies were defined as low frequency (0.04–0.6 Hz) and high frequency (0.6–2.4 Hz) (2, 8, 39). Additionally, we used the low frequency-to-high frequency ratio as an indicator of cardiac autonomic balance. Low frequency and high frequency were expressed as normalized units (n.u.). HRV data analysis was performed using Kubios 3.0.2 software (Finland).

#### Active expiration analysis.

Active expiration was determined as previously described (1, 23). Briefly, 3 segments of respiratory air flow at rest were randomly chosen by a blind operator, and 20 consecutive respiratory cycles were analyzed. Both expiratory time and volume were evaluated. To determine the presence of forced breaths, the expiratory phase was divided into 2 parts: early expiration (E1), corresponding to the initial 50% of the total expiratory time; and late expiration (E2), corresponding to the final 50% of the total expiratory time (1, 23). Increases in the ratio between E2 and E1 expiratory phases (E2/E1) were used as the indicator of active expiration (1). Values of E1 and E2 were obtained by calculations of the area under the first half of the expiratory curve and the area under the second half of the expiratory curve, respectively (1).

#### Cardiorespiratory coupling analysis.

Calculations of coherence between V_T_ and systolic blood pressure (SBP) signals were assessed before and after episodic hypercapnic stimulation using Matlab software (R2106a version, Natick, MA). Auto- and cross-spectral estimates were computed in 10-min, artifact-free recordings using the Welch’s overlapped segment averaging method. A fast Fourier transform (FFT) algorithm was applied to each variable (24). The oscillations in the respiratory signal were taken as the input signal and SBP as the output signal for coherence analysis. The magnitude of the mean square coherence was assessed over a range of 0.1 Hz centered at the frequency of the maximum V_T_ spectral peak in the low-frequency domain (i.e., breathing oscillations) (24).

#### Measurement of arterial blood gases.

Arterial blood gases were measured in conscious, freely moving rats (*n* = 4 per group) using a blood gas analyzer (i‐STAT1 CG8+, Abbott). Under isoflurane (2%), rats were anaesthetized and a vascular access port was placed in the right carotid artery. One week after surgery, animals were put in a whole body plethysmograph and 100 μL of blood were withdrawn at baseline and at 60 min post-EHS. Samples were analyzed immediately, and the volume of blood withdrawn was immediately replaced by an equal volume of sterile saline solution (39) (Supplemental Table S1).

#### Immunofluorescence.

After physiological experiments, rats were deeply anesthetized with urethane (1.8 g/kg iv) then perfused through the ascending aorta with 150 mL of PBS (pH 7.4) followed by 4% paraformaldehyde (0.1 M; pH 7.4) (Sigma, Germany). The brain was removed and stored in the perfusion fixative for 24–48 h at 4°C. Using a vibrating microtome, a series of brain coronal sections (40 μm) were cut and stored in cryoprotectant solution at −20°C (20% glycerol plus 30% ethylene glycol in 50 mM phosphate buffer, pH 7.4) before histological processing. All histochemical procedures were done using free-floating sections (37). The percentage of neurons eliminated after the injection of the SSP-SAP toxin in the RTN (−12.36 to −11.0 to bregma) was determined by immunofluorescence. Tyrosine hydroxylase (TH) was detected using mouse antibody (1: 2,000, Chemicon, Temecula) and Phox2b with a rabbit antibody (1:800, gift from J.-F. Brunet, Ecole Normale Supérieure, Paris, France). These primary antibodies were detected by incubation with appropriate secondary antibodies tagged with fluorescent reporters to reveal TH (goat anti-mouse Alexa 488, Invitrogen, Carlsbad, CA) and Phox2b (donkey anti-rabbit Cy3, Jackson, West Grove, PA). The images were acquired with a high-resolution epifluorescence Leica microscope. The images were quantified using ImageJ software through the content of neurons Phox2b^+^ TH^−^ (National Institutes of Health, Betheseda, MD). Cells were counted using a computer-assisted mapping technique based on the Neurolucida software as previously described (37). The Neurolucida files were exported to the NeuroExplorer software (MicroBright-field, Colchester, VT) to count the various types of neuronal profiles within a defined area. Images were captured with a SensiCam QE 12-bit CCD camera (resolution 1,376 × 1,040 pixels; Cooke, Auburn Hills, MI). IPLab software (Scanalytics, Rockville, MD) was used for merging of color channels in photographs of dual labeling experiments. Immunofluorescence analyses were performed in the same rats on which physiological experiments were performed.

#### Data analysis.

GraphPad Prism 8.0 statistical software (La Jolla, CA) was used to analyze the data. Normal distribution of the data was assessed with the D’Agostino-Pearson test. The statistical significance of the data with normal distribution was evaluated using the one-way ANOVA or two-way ANOVA parametric test, followed by a Sidak post hoc analysis. Statistical significance of nonnormally distributed data was evaluated using nonparametric one-way ANOVA or two-way ANOVA, followed by a Dunn post hoc analysis. The level of significance was defined as *P* < 0.05. Results were shown as mean ±  SE in text and tables and median ± range in figures.

## RESULTS

### 

#### SSP-SAP toxin selectively destroys chemoreceptor neurons of the RTN.

**Fig. 1.**
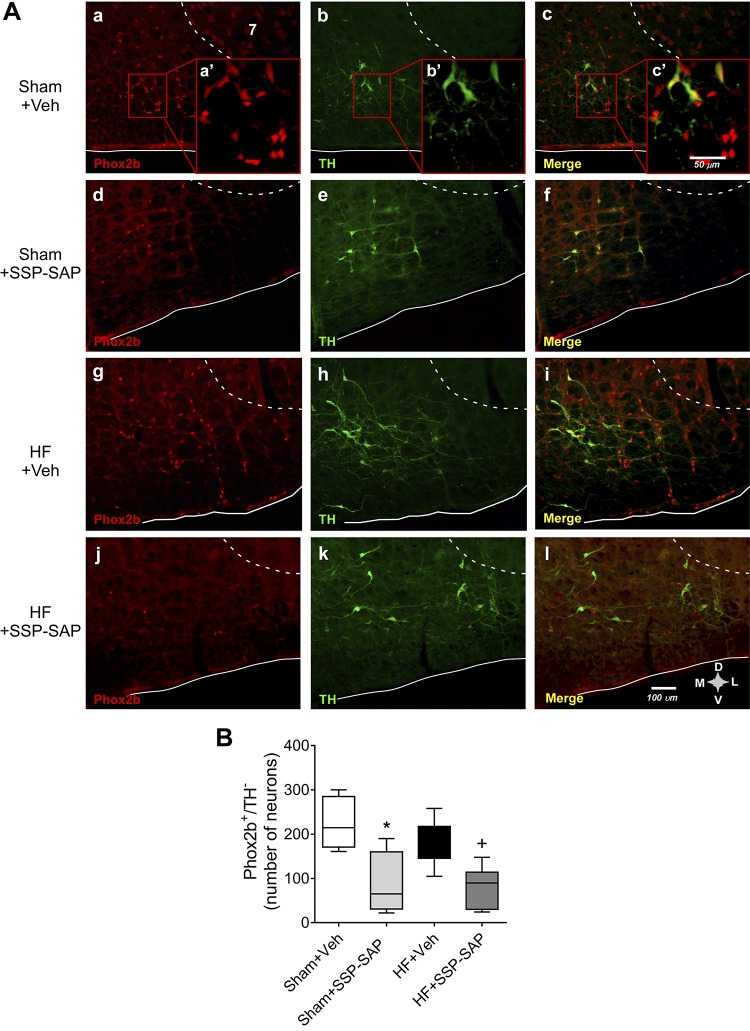
Substance P-conjugated saporin (SSP-SAP) toxin destroys Phox2b-positive neurons but not tyrosine-hydroxylase (TH)-positive neurons within the retrotrapezoid nucleus (RTN). *A*: representative images of histological sections (40 μm) of the ventral surface of the brainstem (bregma level: −11.6 mm) of rats receiving SSP-SAP or vehicle (0.9% NaCl) in the RTN. *B*: quantification of the total number of Phox2b+ TH^−^ neurons in Sham and heart failure (HF) rats treated with vehicle or with the SSP-SAP toxin. One-way ANOVA followed by the Holm-Sidak post hoc test. Box and whiskers represent median ± range. *P < 0.05 vs. Sham+Veh +*P* < 0.05 vs. HF+Veh; *n* = 6 rats per group.

We used substance P-conjugated saporin toxin (SSP-SAP), which destroys Neurokinin 1 (NK1)-positive neurons (expressed by chemoreceptor neurons within the RTN) (38). SSP-SAP toxin or vehicle were injected bilaterally into the RTN of Sham and HF rats according to stereotaxic coordinates. According to previous evidence, Phox2b is predominantly expressed by CO_2_-activated neurons in the RTN (10, 31, 37, 38). Four weeks after treatment, we determined the number of RTN neurons eliminated by SSP-SAP by counting the number of RTN neurons that expressed Phox2b and were devoid of tyrosine-hydroxylase immunoreactivity (Phox2b^+^TH^−^). The percentage of destruction of Phox2b^+^TH^−^ neurons was 45 ± 16% and 50 ± 14% in Sham+SSP-SAP and HF+SSP-SAP rats, respectively, when compared with paired animals that received vehicle injections (Fig. 1). No changes were observed in TH immunoreactivity in the proximity of the injection site. Also, no changes were observed in Phox2b immunoreactivity in other brainstem respiratory sites (i.e., Facial, pre-Bötzinger, Bötzinger).

#### Baseline cardiorespiratory parameters after SSP-SAP treatment.

Baseline hemodynamic and ventilatory data at rest are shown in Table 2. Compared with Sham+Veh and HF+Veh animals, we found that SSP-SAP-treated animals showed no significant change in resting mean blood pressure (100.01 ± 4.39 vs. 96.50 ± 4.95 mmHg, Sham+Veh vs. Sham+SSP-SAP, respectively; 96.01 ± 3.8 vs. 92.01 ± 7.76 mmHg, HF+Veh vs. HF+SSP-SAP, respectively) or heart rate (298.20 ± 13.63 vs. 299.50 ± 14.69 beats/min, Sham+Veh vs. Sham+SSP-SAP, respectively; 331.41 ± 19.33 vs. 285.40 ±19.63, HF+Veh vs. HF+SSP-SAP, respectively). No changes in resting V_T_ or respiratory rate were found between groups (Table 2). Additionally, no significant changes in baseline hemodynamic parameters were observed after EHS (Supplemental Table S2).

**Table 2.** Effect of substance P-saporin toxin on baseline cardiorespiratory parameters

	Sham+Veh (*n* = 6)	Sham+SSP-SAP (*n* = 6)	HF+Veh (*n* = 6)	HF+SSP-SAP (*n* = 6)
Hemodynamic				
SBP, mmHg	124.20 ± 4.57	120.20 ± 6.63	114.60 ± 6.62	115.80 ± 9.78
DBP, mmHg	88.01 ± 4.57	84.83 ± 4.14	87.01 ± 2.86	80.01 ± 6.80
MABP, mmHg	100.01 ± 4.39	96.50 ± 4.95	96.01 ± 3.84	92.01 ± 7.76
PP, mmHg	36.01 ± 2.47	34.67 ± 2.75	27.60 ± 4.78	35.01 ± 3.56
HR, beats/min	298.20 ± 13.63	299.50 ± 14.69	331.41 ± 19.33	285.40 ± 19.63
Ventilatory				
V_T_, mL/100 g	0.31 ± 0.03	0.25 ± 0.01	0.32 ± 0.02	0.26 ± 0.01
R_f_, breaths/min	85.52 ± 2.76	72.58 ± 2.76	85.79 ± 2.70	81.16 ± 2.30
V̇e, mL·100 g^−1^·min^−1^	24.65 ± 1.29	19.35 ± 1.03	22.62 ± 1.78	20.26 ± 1.28

Values are mean ± SE; *n* = 6 rats per group. DBP, diastolic blood pressure; HF, heart failure; HR, heart rate; MABP, mean arterial blood pressure; PP, pulse pressure; R_f_, respiratory frequency; SBP, systolic blood pressure; SSP-SAP, substance P-saporin toxin; V̇e, minute ventilation; V_T_, tidal volume. One-way ANOVA followed by the Holm-Sidak post hoc test.

#### Episodic hypercapnic stimulation triggers ventilatory plasticity in heart failure.

**Fig. 2.**
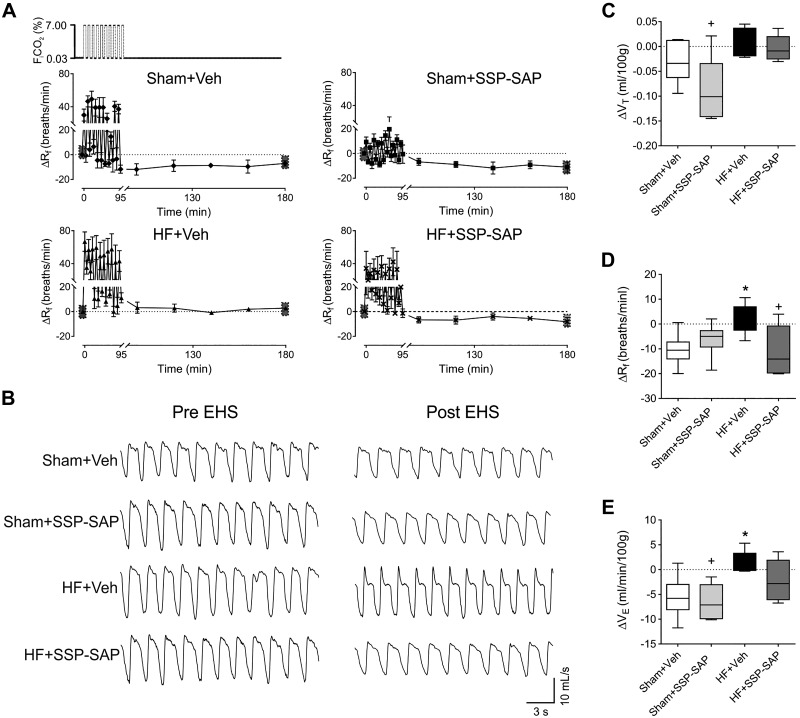
Ventilatory plasticity induced by episodic hypercapnic stimulation in heart failure (HF) is retrotrapezoid nucleus (RTN) chemoreceptor neuron-dependent. *A*: schematic of episodic hypercapnic stimulation (EHS) paradigm (*top panel*). Effect of EHS on ventilation (ΔR_f_) during pre- and post-EHS phases. Note that during the post-EHS phase, Sham+Veh rats showed ventilatory depression, and this effect was absent in HF+Veh rats. Selective ablation of RTN neurons by substance P-conjugated saporin (SSP-SAP) toxin normalizes the post-EHS ventilatory response in HF rats. *B*: representative traces of ventilation pre- and post-EHS in all experimental groups. *C*–*E*: summary data of tidal volume (ΔV_T_), respiratory frequency (ΔR_f_), and minute ventilation (ΔV̇e) during post-EHS phase, respectively. Data represent mean ± SE (*A*) while box and whiskers represent median ± range (*C*–*E*). One-way ANOVA followed by Holm-Sidak post hoc test; *n* = 6 rats per group. *P < 0.05 vs. Sham+Veh; +*P* < 0.05 vs. HF+Veh.

EHS resulted in ventilatory long-term depression in Sham rats, whereas this response was absent in HF rats (Fig. 2). Indeed, HF rats showed augmented ventilation until 90 min after EHS. Both V̇e and R_f_ values were significantly higher in HF+Veh animals compared with Sham+Veh animals after EHS (Fig. 2, *C*–*E*) (ΔV̇e: −5.51 ± 2.10 vs. 1.24 ± 1.35 mL/min 100 g; ΔR_f_: −10.4 ± 3.28 vs. 2.55 ±2.42 breaths/min, respectively). Ablation of RTN neurons blunted EHS-induced changes in ventilation in HF rats (Fig. 2, *A*–*E*). We did not find significant changes in arterial blood gases before or after EHS in all groups (Supplemental Table S1).

**Fig. 3.**
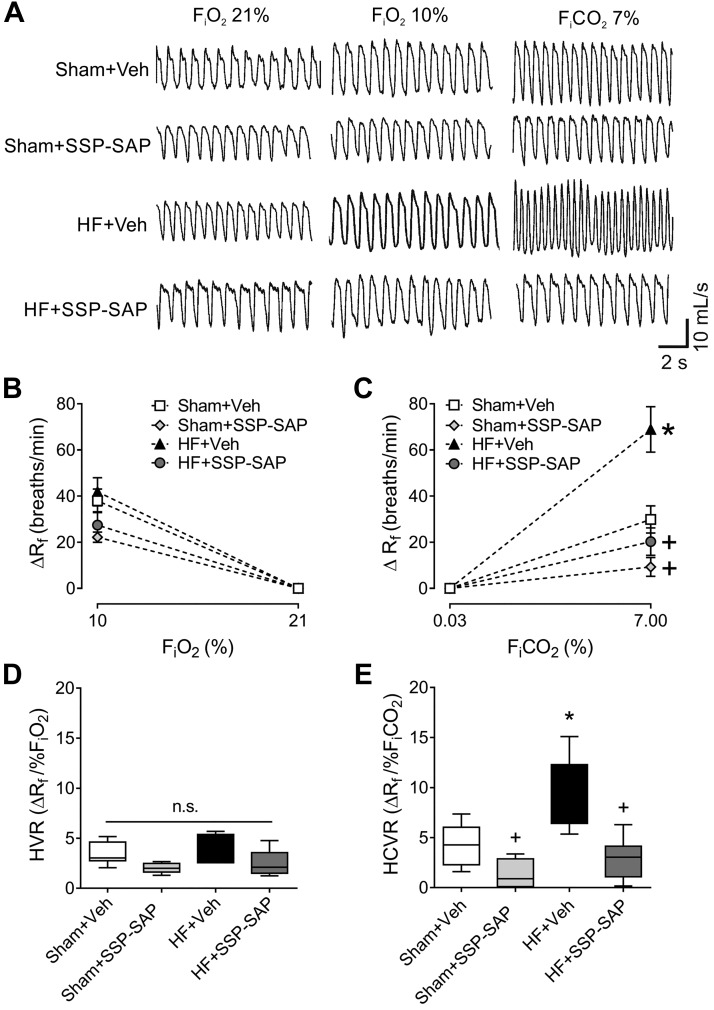
Selective ablation of retrotrapezoid nucleus (RTN) chemoreceptor neurons blunts the hypercapnic ventilatory response (HCVR). *A*: representative traces of ventilation during normoxia ($FI_{O_{2}}$ 21%), hypoxia ($FI_{O_{2}}$ 10%), and hypercapnia (${\text{F}I}_{{\text{CO}}_{\text{2}}}$ 7%). *B* and *C*: respiratory frequency (ΔR_f_) during hypoxia (*B*) and the hypoxic ventilatory response (HVR) (*C*) were not different between groups. *D* and *E*: the respiratory frequency (ΔR_f_) during hypercapnia (*D*) and the hypercapnic ventilatory response (HCVR) (*E*) were increased in heart failure (HF) rats and substance P-conjugated saporin (SSP-SAP) toxin reduced both. Data represent mean ± SE (*B*, *C*), and box and whiskers represent median ± range (*D*, *E*). One-way ANOVA followed by Holm-Sidak post hoc test; *n* = 6 rats per group. *P < 0.05 vs. Sham+Veh; +*P* < 0.05 vs. HF+Veh. n.s., not significant.

To determine whether there was an association between post-EHS, ventilatory plasticity, and central chemoreflex sensitivity in HF, we measured the HCVR (Fig. 3, *A* and *C*). HCVR was significantly higher in HF rats compared with Sham (5.5 ± 0.4 vs. 4.1 ± 0.1 ΔV̇e/${\text{F}I}_{{\text{CO}}_{\text{2}}}$%, respectively), and the enhanced HCVR was reduced by SSP-SAP in HF animals (2.0 ± 0.6 vs. 5.5 ± 0.4 ΔV̇e/${\text{F}I}_{{\text{CO}}_{\text{2}}}$%, HF+Veh vs. HF+SSP-SAP, respectively). We found no significant differences in the HVR in any experimental group (Fig. 3, *B* and *D*).

#### Ablation of RTN chemoreceptor neurons attenuates disordered breathing in heart failure rats.

**Fig. 4.**
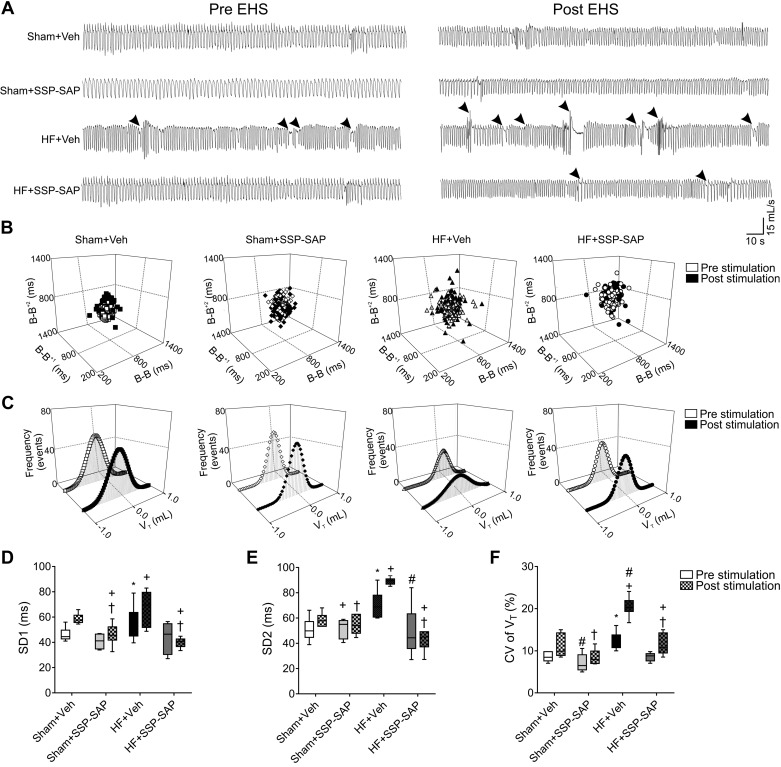
Selective ablation of retrotrapezoid nucleus (RTN) chemosensory neurons prevents ventilatory disturbances elicited by episodic hypercapnic stimulation. *A*: representative traces of ventilation during pre- and post-episodic hypercapnic stimulation (EHS) phases. Arrows point to disturbances in breathing patterns such as apneas/hypopneas. *B* and *C*: representative Poincaré plots and histograms during pre- and post-EHS phases. *D* and *E*: summary data of short-term (SD1) (*D*) and long-term variability (SD2) (*E*), and coefficient of variation (CV) of tidal volume (V_T_) (*F*) during pre- and post-EHS phases. Substance P-conjugated saporin (SSP-SAP) toxin injection in the RTN diminished breath-to-breath and V_T_ amplitude variability in the post-EHS phase in heart failure (HF) rats. Box and whiskers represent median ± range. Two-way ANOVA followed by Holm-Sidak post hoc tests; *n* = 6 rats per group. *P < 0.05 vs. Sham+Veh Pre; #*P* < 0.05 vs. HF+Veh Pre; +*P* < 0.05 vs. Sham+Veh Post; †*P* < 0.05 vs. HF+Veh Post.

Rats with HF displayed marked alterations in resting breathing patterns in normoxia (Fig. 4*A*). HF+Veh rats showed an increase in the breath-to-breath interval variability compared with Sham+Veh animals (Fig. 4, *B*–*F*) (SD2: 71.5 ± 5.4 vs. 53.2 ± 7.9 ms) and in the coefficient of variation of V_T_ (12.9 ± 1.7 vs. 8.7 ± 0.6%, respectively) (Fig. 4, *D* and *F*). SSP-SAP treatment attenuated the alterations observed in resting normoxic breathing patterns in HF rats (Fig. 4). Exposure to EHS exacerbated breathing instability in HF rats but had no effect in other groups (Fig. 4). The coefficient of variation of V_T_ increased ∼2-fold after EHS compared with baseline (12.9 ± 1.7 vs. 20.3 ± 1.5%, HF+Veh pre- vs. post-EHS, *P* < 0.05). SSP-SAP treatment in HF completely abolished the deleterious effects of EHS on breathing stability (Fig. 4).

In addition to abnormalities in ventilatory patterns, we observed a higher incidence of apneas and hypopneas [apnea-hypopnea index, (AHI)] in HF compared with Sham+Veh rats (9.0 ± 1.3 vs. 4.7 ± 0.5 events/h, *P* < 0.05, respectively). SSP-SAP treatment reduced AHI in HF rats (9.0 ± 1.3 vs. 4.3 ± 0.8 events/h, HF+Veh vs. HF+SSP-SAP rats, *P* < 0.05), and the EHS-induced increase in AHI in HF rats was prevented by SSP-SAP treatment in HF (Table 3).

**Table 3.** Effect of substance P-saporin toxin on breathing disorders following episodic hypercapnic stimulation

	Sham+Veh (*n* = 6)		Sham+SSP-SAP (*n* = 6)		HF+Veh (*n* = 6)		HF+SSP-SAP (*n* = 6)	
	Pre	Post	Pre	Post	Pre	Post	Pre	Post
Spontaneous apnea, events/h	3.4 ± 0.4	4.3 ± 0.4	3.3 ± 0.8	4.0 ± 0.7	5.4 ± 0.9*	7.8 ± 0.9+	2.5 ± 1.3	3.7 ± 1.1
Hypopneas, events/h	1.2 ± 0.4	1.6 ± 0.4	0.9 ± 0.2	1.8 ± 0.4	3.7 ± 1.1*	5.8 ± 1.3+	1.7 ± 0.4	2.2 ± 0.7†
Apnea/hypopnea index, events/h	4.7 ± 0.5	6.0 ± 1.1	5.0 ± 0.4	6.0 ± 1.1	9.0 ± 1.3*	14.4 ± 1.3#+	4.3 ± 0.8#	5.5 ± 0.8†
Spontaneous apnea duration, s	3.2 ± 0.3	3.0 ± 0.2	2.9 ± 0.1	3.0 ± 0.2	3.2 ± 0.2	3.1 ± 0.3	3.5 ± 0.3	4.0 ± 0.6
Hypopneas duration, s	2.9 ± 0.2	3.0 ± 0.1	3.0 ± 0.2	2.5 ± 0.1	2.7 ± 0.1	2.5 ± 0.2	2.5 ± 0.4	2.6 ± 0.2
Sights, events/h	12.8 ± 1.2	13.6 ± 1.1	14.8 ± 2.6	18.5 ± 2.5	16.9 ± 2.8	16.8 ± 2.3	14.0 ± 1.2	14.0 ± 0.6
Post sight apnea, events/h	12.0 ± 0.4	12.1 ± 1.9	12.1 ± 0.4	12.0 ± 1.6	14.0 ± 2.1	13.3 ± 2.7	11.0 ± 0.6	10.0 ± 1.2
Post sight apnea duration, s	4.5 ± 0.2	4.4 ± 0.2	3.9 ± 0.2	4.3 ± 0.1	4.2 ± 0.3	3.8 ± 0.2+	4.4 ± 0.2	4.3 ± 0.3†

Values are mean ± SE; *n* = 6 rats per group. AHI, apnoea-hypopnoea index; EHS, episodic hypercapnic stimulation; HF, heart failure; Pre, previous to EHS; Post, 90 min post EHS; SSP-SAP, substance P-saporin toxin. Two-way ANOVA followed by Holm-Sidak post hoc analysis.

* *P* < 0.05 vs. Sham+Veh Pre;

# *P* < 0.05 vs. HF+Veh Pre;

+ *P* < 0.05 vs. Sham+Veh Post;

† *P* < 0.05 vs. HF+Veh Post.

#### Effects of RTN chemoreceptor neuron ablation on EHS-dependent cardiac autonomic imbalance in heart failure.

**Fig. 5.**
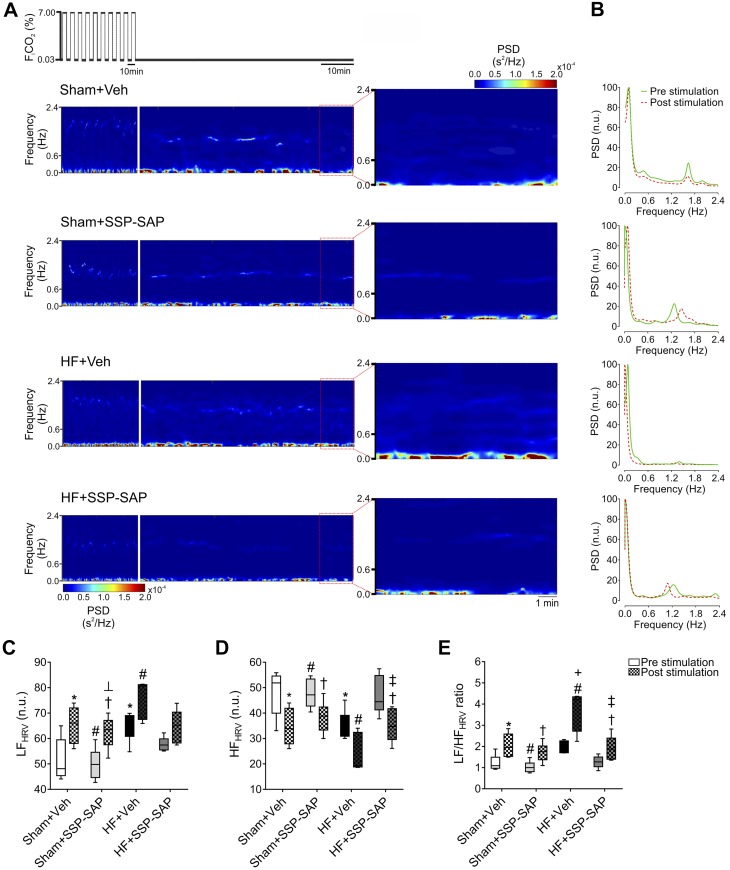
Ablation of retrotrapezoid nucleus (RTN) chemosensory neurons attenuates autonomic imbalance in heart failure (HF) rats. *A*: representative time-varying heart rate variability (HRV) analysis during pre- and post-episodic hyperapnic stimulation (EHS) period in 1 rat per group. Note that HF rats displayed a marked increase in the low-frequency HRV component (0.04–0.6 Hz), and substance P-conjugated saporin (SSP-SAP) toxin injection in the RTN blunted this enhanced sympathetic response during the post-EHS period. *B*: representative power spectral density (PSD) analysis of HRV during pre- and post-EHS phases. *C*–*E*: summary data of low frequency (LF) (*C*), high-frequency (HF, 0.6–2.4 Hz) (*D*), and LF/HF ratio (*E*) during pre- and post-EHS phases. Note that the autonomic imbalance during post-EHS phase was blunted by SSP-SAP toxin delivery into the RTN. Box and whiskers represent median ± range. Two-way ANOVA followed by Holm-Sidak post hoc analysis; *n* = 6 rats per group. *P < 0.05 vs. Sham+Veh Pre; #*P* < 0.05 vs. HF+Veh Pre; ┴*P* < 0.05 vs. Sham+SSP-SAP Pre; ‡*P* < 0.05 vs. HF+SSP-SAP Pre; +*P* < 0.05 vs. Sham+Veh Post; †*P* < 0.05 vs. HF+Veh Post. n.u., normalized units.

At baseline, HF rats displayed an increase in the low-frequency component of HRV (LF_HRV_) and a decrease in the high-frequency component of HRV (HF_HRV_) compared with Sham rats (Fig. 5). These effects were attenuated by ablation of RTN chemoreceptor neurons in HF rats (Fig. 5, *A*–*E*). After EHS, changes in cardiac autonomic balance were observed in both Sham and HF rats and were characterized by additional increases in the LF_HRV_ component and a decrease in the HF_HRV_ component (Fig. 5, *A*–*E*). Accordingly, the LF_HRV_-to-HF_HRV_ ratio, an indirect measure of cardiac autonomic balance, was significantly increased (*P* < 0.05) by EHS in Sham+Veh (1.1 ± 0.1 vs. 2.2 ± 0.2, pre- vs. post-EHS;) and in HF+Veh rats (2.0 ± 0.2 vs. 3.6 ±0.7, pre- vs. post-EHS;); however, the effect of EHS on cardiac autonomic imbalance was larger in HF animals (Fig. 5*E*). Selective ablation of RTN chemoreceptor neurons significantly attenuated the EHS-induced changes in cardiac autonomic balance in HF (Fig. 5).

#### Active expiration in heart failure rats and effects of RTN chemoreceptor neuron ablation.

**Fig. 6.**
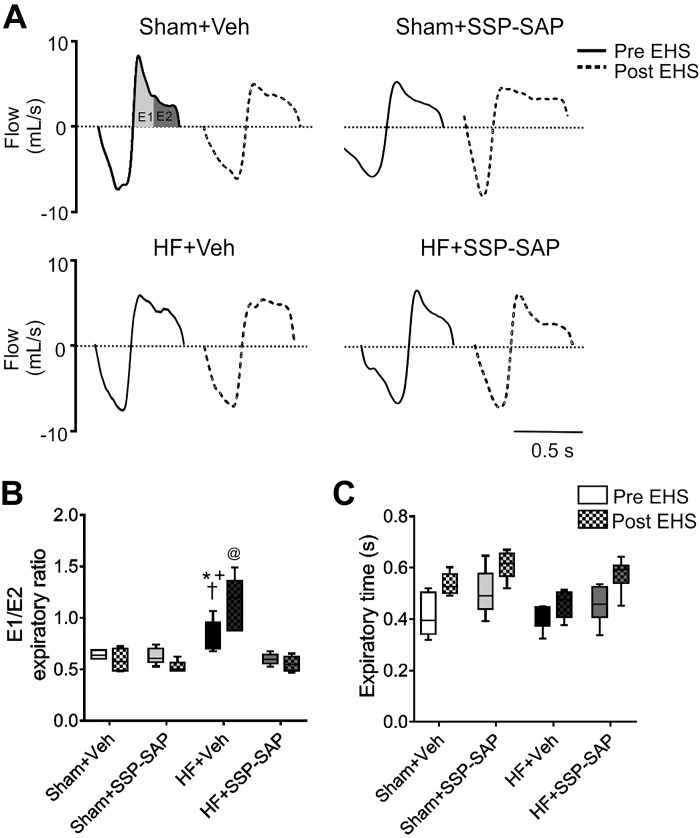
Episodic hypercapnic stimulation further increases active expiration in heart failure (HF) rats. *A*: representative traces of one breathing cycle pre- (continuous traces) and post- (segmented traces)-episodic hypercapnic stimulation (EHS) in 1 Sham+Veh rat, 1 Sham+SSP-SAP-treated rat, 1 HF+Veh rat, and 1 HF+SSP-SAP-treated rat. In HF rats, the early expiratory flow (E1) was reduced, whereas the late expiratory flow (E2) was increased compared with Sham rats. *B*: summary data showing E2/E1 ratio. *C*: summary of expiratory time obtained in 20 consecutive respiratory cycles in all groups, pre- and post-EHS. Box and whiskers represent median ± range. Two-way ANOVA followed by post hoc analysis of Holm-Sidak; *n* = 6 rats per group. *P < 0.05 vs. Sham+Veh; +*P* < 0.05 vs. Sham+SSP-SAP; †*P* < 0.05 vs. HF+SSP-SAP; @*P* < 0.05 vs. HF+Veh post-EHS.

Compared with Sham rats, HF rats displayed active expiration in normoxia as evidenced by an increase in the early-to-late expiration ratio (E2/E1) (0.89 ±0.19 vs. 0.68 ± 0.09 HF+Veh vs. Sham+Veh, respectively, Fig. 6, *A* and *B*). In HF rats, partial elimination of RTN chemoreceptor neurons nearly eliminated active expiration, mainly by decreasing the late expiratory phase without significant changes in the early expiratory phase (E2: 0.05 ± 0.01 vs. 0.08 ± 0.01 mL, HF+Veh vs. HF+SSP-SAP, respectively). After EHS, active expiration was exacerbated in HF rats, as evidenced by the large increase in E2/E1 (0.89 ± 0.19 vs. 1.24 ± 0.24, HF+Veh pre-EHS vs. HF+Veh post-EHS, respectively, Fig. 6, *A* and *B*). The effect of EHS on the increase of E2/E1 in HF rats was dependent on the integrity of RTN chemoreceptor neurons because SSP-SAP treatment in HF rats resulted in a significant decrease in E2/E1 (1.24 ± 0.19 vs. 0.55 ± 0.07, HF+Veh post-EHS vs. HF+SSP-SAP post-EHS, respectively, Fig. 6,* A* and *B*). No significant changes in the total expiratory time were observed in any of the experimental groups (Fig. 6*C*).

#### Ablation of RTN chemoreceptor neurons attenuates EHS-dependent cardiorespiratory coupling in rats with heart failure.

**Fig. 7.**
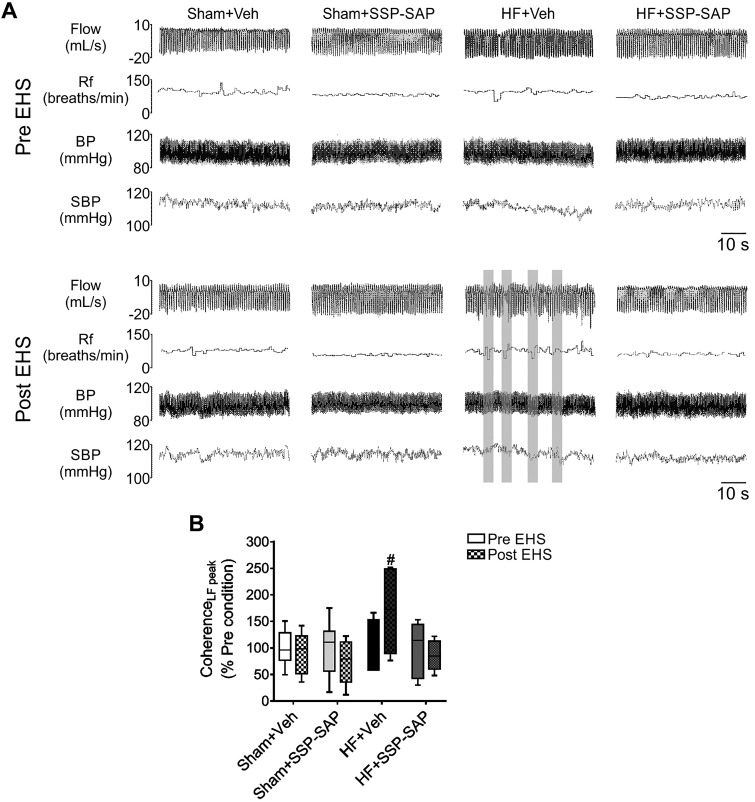
Episodic hypercapnic stimulation-induced respiratory-cardiovascular coupling in heart failure depends on intact retrotrapezoid nucleus (RTN) chemoreceptor neurons. *A*: representative traces of respiratory flow, respiratory frequency (R_f_), blood pressure (BP), and systolic blood pressure (SBP) in 1 rat per group during pre- and post-episodic hypercapnic stimulation (EHS) phase. Segments where coupling between ventilatory and cardiovascular signals was observed are highlighted (gray). *B*: summary data of coherence analysis. Note that following EHS, heart failure (HF) rats displayed an increased coherence between tidal volume (V_T_) oscillation and SBP, and this was blunted in HF rats treated with substance P-conjugated saporin (SSP-SAP) toxin. Box and whiskers represent median ± range. Two-way ANOVA followed by Holm-Sidak post hoc analysis; *n* = 6 rats per group. #*P* < 0.05 vs. HF+Veh Pre-EHS. LF, low frequency.

Coupling between cardiac autonomic function and ventilation was determined by calculating the coherence between oscillations in the V_T_ and SBP. Cardiorespiratory coupling was increased (i.e., increased coherence) by EHS only in HF rats (Fig. 7), and SSP-SAP treatment blunted the EHS-induced increase in cardiorespiratory coupling in HF animals (Fig. 7). EHS had no effects on cardiorespiratory coupling in Sham+Veh or Sham+SSP-SAP-treated rats.

## DISCUSSION

The main aim of this study was to assess whether acute episodic stimulation of chemoreceptors with hypercapnia in the setting of volume overload HF could elicit ventilatory plasticity, leading to the onset of irregular breathing patterns and autonomic imbalance (3, 7, 11, 18). We have previously proposed that periodic stimulation of chemoreflex pathways may play a major role in the maladaptive cardiorespiratory changes associated with HF (40). In this study, we show that episodic stimulation of the central chemoreflex elicits ventilatory long-term depression in normal rats, which was not observed in HF+Veh animals. Importantly, this phenomenon was associated with the development of irregular breathing patterns, increased AHI, and exacerbation of cardiac autonomic imbalance in HF rats. Furthermore, our data show that these responses are largely dependent on RTN chemoreceptor neurons, since their selective destruction using SSP-SAP blunted ventilatory changes and EHS-induced breathing/autonomic disturbances in HF animals. Ventilatory long-term depression in response to hypercapnia in healthy rats has previously been reported (4); however, those experiments were performed in anesthetized, vagotomized, and mechanically-ventilated animals (4). This is the first study to address the effects of EHS on ventilatory patterns and autonomic function in unrestrained rats and the first to address these parameters in the setting of volume overload HF. The precise mechanisms responsible for EHS-induced long-term depression of ventilation have not been thoroughly studied; however, our results suggest that central chemoreceptors play a pivotal role in this phenomenon. Indeed, the normal long-term depression of ventilation observed after EHS was absent in HF+Veh rats, concomitant with enhanced CC sensitivity. More importantly, ablation of RTN chemoreceptor neurons attenuated abnormal ventilatory responses to EHS in HF rats.

### 

#### Central chemoreceptors and heart failure.

There are numerous putative sites for central chemoreception in the brain; however, the medullary RTN is thought to be a primary region. Chemoreceptor neurons within the RTN are activated by changes in cerebrospinal fluid levels of CO_2_/H^+^ and send projections to the rCPG (21, 32, 40) and the RVLM (28, 35), the latter being considered a major nodal point for integration of sympathetic drive. Upon activation, the RTN initiates reflex increases in respiratory rate and sympathoexcitation (14). Additionally, activation of the RTN induces cardiorespiratory coupling, a phenomenon that has been linked to chronic sympathoexcitation (13). In addition to their contribution to CO_2_ homeostasis, there is evidence that central chemoreceptor function is altered in disease states. Indeed, augmented central chemoreflex sensitivity, breathing disturbances, and autonomic dysfunction are commonly observed in patients with HF (11, 19, 29, 40, 42). Clinical studies in HF patients show a positive correlation between CO_2_ chemosensitivity and AHI (36), and that hypercapnic stimulation elicits an increase in sympathetic nerve activity in HF patients but not in healthy ones. Furthermore, our previous studies in animal models of HF strongly support the notion that enhanced central chemoreflex sensitivity contributes to the development of disordered breathing and sympathoexcitation (39). Based on these findings, we hypothesized that acute episodic stimulation of central chemoreceptors, which may occur clinically as a result of disordered breathing patterns in HF, may exacerbate ventilatory instability in part via induction of ventilatory plasticity.

#### Central chemoreceptors and disordered breathing patterns in heart failure.

From a clinical perspective, ~40% of HF patients have central sleep apnea, combined with a higher HCVR (18, 36). The proposed relationship between these two phenomena is a high resting minute-ventilation resulting in CO_2_ ‘wash-out’ from central chemosensitive areas and closer resting proximity to the apneic threshold (38). We observed that EHS in HF rats resulted in augmented ventilation, in contrast to Sham rats, in which EHS elicited ventilatory long-term depression. Of note, we found that selective ablation of RTN chemoreceptor neurons using SSP-SAP in HF rats prevented the effects of EHS but did not have a similar effect in Sham rats. In addition, our results show that SSP-SAP injection into the RTN reduced breathing instability at rest in HF rats under baseline conditions. These results suggest a prominent role for RTN chemoreceptor neurons in shaping resting breathing patterns in HF before and after hypercapnic challenges. These findings confirm and extend previous reports showing that RTN chemoreceptor neurons regulate normoxic ventilation (10, 31, 37, 38, 41). Together, these results suggest a crucial role for RTN chemoreceptor neurons in the regulation of breathing patterns in HF, suggesting that RTN neurons are a plausible therapeutic target to reduce resting hyperventilation and decrease AHI in HF. Future studies should focus on the precise mechanisms underlying RTN chemoreceptor neurons and regulation of breathing in HF.

#### Central chemoreceptors and cardiac autonomic dysfunction in heart failure.

Three major brain nuclei have been linked to sympathoexcitation in HF: the hypothalamic paraventricular nucleus (PVN), the nucleus of the solitarii tract (NTS), and the RVLM (19, 42), with the RVLM being the major regulatory area for sympathetic outflow (13, 33). We have previously shown that enhanced central chemoreflex gain is associated with increased Fos B labeling in the RVLM of HF rats, suggesting a link between central chemoreceptors and chronic neuronal activation in presympathetic regions of the brainstem (39). Given that putative connections exist between the RTN and the RVLM (28, 35), we hypothesized that EHS of central chemoreceptors would enhance cardiac sympathetic tone. In this study, EHS increased cardiac sympathetic tone in both healthy and HF conditions, but the effect was larger in rats treated with vehicle compared with those that received SSP-SAP injections. Importantly, SSP-SAP injection into the RTN of HF rats attenuated the higher cardiac sympathetic tone elicited by EHS. Furthermore, SSP-SAP not only reduced the EHS-induced sympathetic response in HF, but also normalized baseline cardiac autonomic imbalance (LF_HRV_-to-HF_HRV_ ratio) before application of EHS. Importantly, SSP-SAP treatment did not affect catecholaminergic neurons surrounding the RTN in either Sham or HF animals, which play an important role in regulating sympathetic outflow to the heart (28, 35); thus, improvements in autonomic control observed in HF+SSP-SAP are unlikely to be related to an off-target effect of SSP-SAP treatment. Taken together, these results suggest that RTN chemoreceptor neurons play a role in cardiac autonomic regulation in HF, probably by regulating stimulation of presympathetic neurons in the RVLM. Although RTN neurons make synaptic connections with NTS neurons (13, 28, 35), previous studies indicate that acute inhibition of the NTS has no significant effects on sympathetic activity or central chemoreflex function (28). Therefore, a putative RTN-RVLM connection as well as its role in HF progression deserves further investigation.

Active expiration is commonly associated with a concomitant increase in sympathetic outflow (1). Indeed, the presence of both active expiration and sympathoexcitation has been linked to cardiorespiratory dysfunction (30). We found that HF rats displayed active expiration at rest during eupneic conditions, suggesting the recruitment of abdominal muscles during resting breathing. Importantly, it has been shown that stimulation of RTN neurons results in activation of expiratory muscles and active expiration (17). In our studies we found that SSP-SAP treatment abolished active expiration in HF rats in normoxic conditions. These results strongly support the notion that RTN chemoreceptor neurons are required for the maintenance of active expiration in the setting of volume overload HF. Future studies using EMG recordings are needed to fully assess the presence and contribution of the RTN on active expiration in high-output HF (17). In addition to our observations of the effects of HF on active expiration during resting breathing, we found that EHS induces a further increase in late-expiratory flows in HF rats. This was dependent on RTN chemoreceptor neurons because SSP-SAP completely eliminated the EHS-induced active expiration. Lastly, we showed that EHS-induced active expiration occurs in tandem with cardiac autonomic imbalance in HF rats. These results suggest that RTN chemoreceptor neurons may serve as a nodal point for the entrainment of respiratory and cardiovascular function in HF rats. Importantly, alterations in respiratory-cardiovascular coupling has been proposed to underlie the pathophysiology of oscillatory breathing in HF (40). In this study, respiratory-cardiovascular coupling was observed during the onset of breathing disorders, which was partially mediated by RTN chemoreceptor neurons because SSP-SAP decreases respiratory-cardiovascular coupling in HF rats. Nevertheless, we cannot rule out the contribution of sympathoinhibitory reflexes elicited by lung stretch receptors (12) in the genesis of disordered breathing in HF. Further studies should focus on the role of pulmonary receptors in the development of altered breathing patterns and autonomic imbalance in HF.

#### Strengths and limitations.

To date, the vast majority of studies in the field of chemoreflex function and heart failure (HF) pathophysiology have been done in reduced ejection fraction models (≤35% LVEF) (6). These models are characterized by overt reductions in tissue perfusion, including both central and peripheral chemosensory areas (7, 9, 25). To our knowledge, this is the first study showing that RTN chemoreceptor neurons play a role in cardiorespiratory alterations after EHS in the setting of HF without the confounding effect of chronic reductions in tissue perfusion because previous evidence from our laboratory showed volume overload HF rats displayed no chronic decrease in LVEF at 8 wk post-HF induction (7). From a translational perspective, volume overload HF recapitulates some but not all characteristics of human HF with preserved ejection fraction (43). Although there are obvious differences between the etiology of human HF and the experimental volume overload model used in this study, it is important to note that both preserved ejection fraction HF in humans and experimental volume overload HF have comparable levels of disordered breathing and sympathetic activation. These two important hallmarks of human HF are positively correlated with disease progression and poor prognosis.

Despite the fact that SSP-SAP injections within the RTN region primarily result in the ablation of RTN chemoreceptor neurons (37, 38), some studies have shown off-target effects such as reduced NK1R immunoreactivity in C1 neurons in the vicinity of the injection site (37). However, when administered at a dose that kills 90% of Phox2b+ RTN neurons, the toxin spares nearby C1 catecholaminergic, serotonergic, and cholinergic neurons (37). However, considering that C1 neurons constitutively express substance P receptors (13), it is plausible that injections of SSP-SAP into the RTN may target C1 neurons as well. Nevertheless, our data showing that Sham+SSP-SAP-treated rats display similar levels of cardiac sympathetic outflow compared with Sham+Veh rats strongly suggest that there was no physiological effect of SSP-SAP on sympathetic control areas in close proximity to the injection site.

Interestingly, we found that Sham rats continue to display a post-EHS hypoventilation even after ablation of RTN Phox2b+ neurons. This result strongly suggests that, contrary to what is observed in HF rats, other structures besides the RTN contribute to the hypoventilatory response after EHS in healthy rats. Indeed, raphe adrenergic and serotoninergic neurons have been linked to ventilatory long-term depression in healthy animals (4, 44). Additionally, we wish to acknowledge that peripheral carotid body chemoreceptors, which are able to elicit a chemoreflex response during hypercapnic stimulation, may also play a role in EHS-induced hypoventilation observed in sham animals. With that said, we observed no differences in the HVR between HF and Sham rats. Discrepancies from our previous studies showing a reduction in HVR in HF rats (40) are likely due to the marked difference in the protocol used to assess HVR. In previous studies, we used only 2–3 min of hypoxic gas stimulation (40) compared with the 10-min hypoxic exposure used in the present study. However, it is important to note that we did not observe a potentiation of the HVR in the present or previous studies. Nevertheless, we cannot preclude the possibility that tonic afferent drive from the carotid bodies modulates the central chemoreflex and contributes to altered breathing patterns and cardiac autonomic dysfunction in the setting of volume overload HF. Future studies are needed to precisely define the role of the carotid body on cardiorespiratory adjustments in volume overload HF before and after EHS.

In summary, our results indicate that EHS triggers ventilatory plasticity in healthy rats, characterized by long-term ventilatory depression. Interestingly, this physiological response to EHS was blunted in volume overload HF rats. Furthermore, EHS exacerbates breathing instability and the incidence of breathing disorders in HF. Additionally, EHS worsens cardiac autonomic imbalance in HF rats. Importantly, we found that RTN chemoreceptor neurons play a seminal role in EHS-induced ventilatory plasticity, disordered breathing, and exacerbation of cardiac autonomic imbalance in rats with volume overload HF because partial ablation of these neurons restores normal cardiorespiratory responses to EHS in HF rats.

## GRANTS

This work was supported by Fondo de Desarrollo Científico y Tecnológico Fondecyt 1180172 (to R. Del Rio), the Basal Center of Excellence in Aging and Regeneration (AFB 170005), and the special grant “Lithium in Health and Disease” from the Sociedad Química y Minera de Chile (SQM). This work was also supported by the São Paulo Research Foundation (FAPESP; grants: 2015/23376-1 and 2016/22069-0 to T. S. Moreira; 2016/23281-3 to A. C. Takakura).

## DISCLOSURES

No conflicts of interest, financial or otherwise, are declared by the authors.

## AUTHOR CONTRIBUTIONS

R.D.R. conceived and designed research; H.S.D., D.C.A., C.T., K.V.P., K.G.S., E.D.-J., C.L., A.A.-A., and J.N.S. performed experiments; H.S.D., D.C.A., C.T., K.V.P., K.G.S., E.D.-J., C.L., A.A.-A., and J.N.S. analyzed data; H.S.D., D.C.A., C.T., H.D.S., A.C.T., T.S.M., N.J.M., and R.D.R. interpreted results of experiments; H.S.D., D.C.A., C.T., K.G.S., J.N.S., A.C.T., and T.S.M. prepared figures; H.S.D., D.C.A., C.T., C.L., A.A.-A., H.D.S., N.J.M., and R.D.R. drafted manuscript; H.S.D., D.C.A., C.T., K.V.P., K.G.S., E.D.-J., H.D.S., A.C.T., N.J.M., and R.D.R. edited and revised manuscript; H.S.D., D.C.A., C.T., K.V.P., K.G.S., E.D.-J., C.L., A.A.-A., H.D.S., J.N.S., A.C.T., T.S.M., N.J.M., and R.D.R. approved final version of manuscript.
